# Assessment of the anti-biofilm effect of UV-C irradiation (254 nm) against healthcare associated infections related microorganisms

**DOI:** 10.3389/fmicb.2025.1570334

**Published:** 2025-07-22

**Authors:** Francesco Palma, Marta Díaz-Navarro, Andrés Visedo, Pablo Sanz-Ruíz, Giorgio Brandi, Giuditta Fiorella Schiavano, María Guembe

**Affiliations:** 1 Department of Biomolecular Sciences, University of Urbino Carlo Bo, Urbino, Italy; 2 Instituto de Investigación Sanitaria Gregorio Marañón, Madrid, Spain; 3 Department of Clinical Microbiology and Infectious Diseases, Hospital General Universitario Gregorio Marañón, Madrid, Spain; 4 Medicine Department, School of Medicine, Universidad Complutense de Madrid, Madrid, Spain; 5 Department of Orthopaedic Surgery and Traumatology, Hospital General Universitario Gregorio Marañón, Madrid, Spain; 6 Department of Humanities, University of Urbino Carlo Bo, Urbino, Italy

**Keywords:** UV-C, biofilm, hospital surface, disinfection, healthcare-associated infection

## Abstract

**Introduction:**

Biofilm-related Multidrug Resistance (MDR) is a major problem in healthcare-associated infections (HAI). Hospital surface decontamination is essential to ensure the safety of patients and to eliminate the dissemination of MDR pathogens. New eco-friendly decontamination technologies, such as UV-C irradiation, are only gaining popularity now, but their use against the biofilm of common microorganisms causing HAI has not been properly assessed. We aimed to assess the efficacy of UV-C irradiation (254 nm) in a 2-phase study by assessing its anti-biofilm effect against sessile cells from microorganisms of hospital interest.

**Methods:**

The following strains were tested: methicillin-susceptible *Staphylococcus aureus* (MSSA) (ATCC 29213), methicillin-resistant *Staphylococcus aureus* (MRSA) (ATCC 43300), *Escherichia coli* ATCC 25922, *Pseudomonas aeruginosa* ATCC 15442, and *Candida albicans* (ATCC 14053), and a clinical strain of methicillin-resistant *Staphylococcus epidermidis*. First, the tested strains' UV-susceptibility was evaluated through irradiation tests on plates using different UV doses, considering both planktonic and 24 h-biofilm states. Second, the anti-biofilm effect of UV-C was evaluated on stainless steel discs contaminated with a 24 h-biofilm of each strain.

**Results:**

With a UV dose of 946.7 mJ/cm^2^, the UV-C irradiation on MSSA ATCC 29213, MRSA ATCC 43300, and MRSE biofilm showed a log_10_ reduction of 4.34 ± 0.70, 4.70 ± 0.60, and 4.85 ± 0.98, respectively, while *C. albicans* ATCC 14053 showed higher UV-resistance in 24 h-biofilm state, being the log_10_ reduction of 3.17 ± 0.08. Against Gram negative bacteria biofilm, a UV dose of 467.8 mJ/cm^2^ was enough to achieve a microbial titer < 1 CFU/mL. Regarding the 24 h-biofilm on discs, a log_10_ reduction >3 logs was achieved with all microorganisms applying a UV dose of 946.7 mJ/cm^2^.

**Conclusion:**

The application of UV-C irradiation could be a valid additional approach in the management of biofilm HAI.

## 1 Introduction

In recent years, heightened attention has been directed toward the cleaning procedures of surfaces and instruments, particularly in environments where vulnerable individuals are present, such as in nursing homes and hospitals (Pereira et al., 2017; Reddy et al., 2021). Optimal disinfection is particularly important in operating rooms, where the use of contaminated operating tools would significantly increase the risk of post-operative complications (CDC and ICAN, 2019). About 60 to 70% of nosocomial infections (NI) are linked to medical devices, and it has been estimated that between 50 and 70% of nosocomial infections are caused by biofilm formation on implanted medical devices (Yasir et al., 2018; Asker et al., 2021). Biofilm-related multi-drug resistance is a major problem in the nosocomial infection context (Assefa and Amare, 2022). Hospital surface decontamination is essential to ensure the safety of staff and patients and crucial to eliminate the dissemination of multidrug-resistant (MDR) pathogens (dos Santos and de Castro, 2021). The ongoing problems related to multiple-antibiotic resistance in clinically important bacterial strains in hospital settings and the potential increase in resistance to disinfectants, the use of which is increasing in the community, increase patients' risk of infection (CDC and ICAN, 2019). Specifically, the production and sedimentation of potential biocides residual on inanimate surfaces by chemical disinfectants or their inappropriate application may contribute to the emergence and dissemination of antibiotic resistance in bacteria (Li et al., 2023; Maillard and Pascoe, 2023).

As an alternative to chemical disinfectants, disinfection by UV irradiation has been re-evaluated in recent years. UV-based disinfection technology is based on exposure to UV radiation of wavelengths ranging from 100 to 400 nm. Compared to chemical disinfection methods, UV disinfection presents many advantages. UV irradiation does not release toxic by-products on the treated surface and it offers a rapid and non-destructive disinfection method (Palma et al., 2022, 2024). However, some studies reported that in some microorganisms, UV irradiation and the use of antibiotics would seem to stimulate an SOS response, i.e., a bacterial survival mechanism against genotoxic damage, increasing the mutation rate and sometimes contributing to antibiotic resistance (Beaber et al., 2004; Krishna et al., 2007). In addition, another significant disadvantage of the use of chemical products is their reduced eco-sustainability and their high environmental impact, a risk factor as significant for human health as for the balance of ecosystems.

As known, the microbicidal effect of UV-C is due to its ability to break the hydrogen bonds between thymine and adenine, inducing dimerization between two consecutive thymine residues, thus forming cyclobutane pyrimidine dimers (CPD). The presence of this CPD inhibits the DNA polymerase activity, causing a slowdown in cell replication and cell death. However, it must be considered that some microorganism's features can reduce the UV-C disinfection efficacy, such as: the quantity of thymine bases in the genetic material; the ability to form endospores or biofilms; the presence of UV-absorbing pigments or compounds that can protect microorganisms against UV rays (Suma et al., 2020). The presence of photolyase enzymes, such as DNA photolyase, that can bind the CPD covalent bonds using the visible light energy (Marizcurrena et al., 2017).

It must be underlined that the exposure time and the distance of the UV lamp from the material to be sanitized considerably affect the rate of microbial abatement. The material and design of surfaces can also influence the germicidal action of the light. As reported in different studies (Ariani, 2015; Ariani et al., 2015; Malateaux et al., 2021), the permeability and irregularity of surface materials make them difficult to clean, consequently increasing their susceptibility to microbial colonization. In addition, the composition of the material can also change the adhesion of microorganisms by forming biofilms that are resistant to disinfection processes (Corrêa et al., 2017).

The process of biofilm formation starts when microorganisms attach to surfaces and aggregate in a self-produced extracellular polymeric substance (EPS), offering protection against external environmental factors (Mirghani et al., 2022). Bacteria growing within biofilms were found to be more resistant to treatment with antimicrobial agents than planktonic cells (Luo et al., 2022). However, how this resistance affects the outcome of the UV irradiation on biofilms is not clearly known. In recent years, UV technology has developed greatly, which has increased its applicability in various contexts and environments.

An important aspect that must be considered is that most microorganisms causing nosocomial infections, such as the microorganisms considered in this study, are biofilm producers, and this significantly increases their resistance to disinfection treatments, including UV irradiation (Corrêa et al., 2017; de Carvalho, 2017; Roy et al., 2018; Cangui-Panchi et al., 2022). To date, there are only a few studies in which the efficacy of UV-C irradiation on biofilms of major microorganisms of hospital interest has been evaluated, and those few that are considered biofilms in the process of formation and not a formed biofilm (Chen et al., 2020; Torkzadeh et al., 2021; Mariita et al., 2022).

Therefore, we aimed to assess the efficacy of UV-C irradiation (254 nm) in a 2-phase study by assessing its anti-biofilm effect against sessile cells of hospital-interest microorganisms.

## 2 Material and method

### 2.1 Bacterial strains and growth condition

In this study, five ATCC strains, namely, methicillin-susceptible *S. aureus* ATCC 29213 (MSSA), methicillin-resistant *S. aureus* ATCC 43300 (MRSA), *E. coli* ATCC 25922, *Pseudomonas aeruginosa* ATCC 15442, and *C. albicans* ATCC 14053, were selected. Moreover, a clinical strain of methicillin-resistant *Staphylococcus epidermidis* (MRSE), showing optimal biofilm production (by crystal violet assay) from the microbiology laboratory, was also selected. Its ability to form biofilms on both plates and steel discs has been previously evaluated (Márquez-Gómez et al., 2023). MSSA ATCC 29213, MRSA ATCC 43300, *S. epidermidis*, and *P. aeruginosa* ATCC 15442, were growth on blood agar plates; *E. coli* ATCC 25922 on Agar MacConkey (MCK), and *C. albicans* ATCC 14053 on Chromagar^TM^ Candida.

### 2.2 Test microorganism suspension preparation

In both phases of the study, bacterial suspensions of MSSA, MRSA, MRSE, *P. aeruginosa, and E. coli* at a concentration of 10^8^ CFU/mL (0.5 McFarland) and a yeast suspension of *C. albicans* at a concentration of 10^6^ CFU/mL (0.35 McFarland) were used. The bacterial suspensions were prepared in broth media (TSB for both *S. aureus* strains, TSB+1% glucose for *S. epidermidis*, LB for *E. coli*, and BHI for *P. aeruginosa*) previously incubated at 37°C for 18 h under agitation, while the yeast suspension was prepared in Roswell Park Memorial Institute (RPMI). All microorganism overnight cultures were washed twice with phosphate-buffered saline (PBS) and resuspended in the same growth broth medium, except *C. albicans* overnight cultures, which were prepared in Yeast Peptone Dextrose Broth (YPD). All microorganism suspension concentrations were experimentally confirmed by plate culture.

### 2.3 Microbial susceptibility evaluation in planktonic and sessile stages

To perform this 2-phase study, a low-pressure mercury UV-C lamp (254 nm, Philips, 11W) was used, and the UV-doses were measured using a radiometer (HD2102.2, LP 471 UV-C). During the tests, the UV-C lamp was positioned 50 cm from the sample. In the first phase of the study, irradiation tests on plates using different UV doses were assessed to evaluate the UV-C susceptibility of each tested strain, considering both planktonic and 24 h-biofilm states. The biofilm-producing capacity of the strains considered was previously assessed by the crystal violet (CV) biomass assessment method (OD > 0.5) (Díaz-Navarro et al., 2023; Márquez-Gómez et al., 2023). The UV doses used for each experimental condition are reported in Table 1.

**Table 1:** UV doses (mJ/cm^2^) used against planktonic and sessile cells.

**Experimental condition**	**Wavelength (nm)**	**UV dose (mJ/cm^2^)**
Planktonic		12.8
	254	37.4
		83.8
24 h-biofilm		228.6
	254	467.8
		946.7

**Planktonic**: To perform planktonic exposure tests, 100 μl of each bacteria or yeast suspension was spread on different solid mediums, based on the test microorganism considered. The kind of medium used in this study phase for each microorganism is reported in paragraph 2.1. Subsequently, the planktonic cells were treated with three different UV doses (shown in Table 1). After incubation at 37 °C for 24 h, the CFU/mL was calculated.

**Sessile cell**: To form a biofilm, 100 μl of each bacteria or yeast suspension was inoculated into the 96-well cell culture microtiter plates and incubated at 37 °C for 24 h. Each biofilm was washed gently three times with PBS to remove any non-adhered cells and, before the UV treatment, the PBS was removed. Once the biofilms on the wells were dried, the wells were treated with three different UV doses, which are shown in Table 1. After the UV treatment, the content of the wells was scraped, resuspended in PBS, and cultured on a solid medium in serial dilutions. Plates were then incubated for 24 h at 37°C, and then the CFU/mL was calculated.

All data were expressed as log_10_ CFU/mL, and the log_10_ reductions were calculated considering as control the UV-untreated samples. All the experiments were replicated three independent times.

### 2.4 Evaluation of UV-C anti-biofilm effect on stainless steel surface

In the second phase of the study, the anti-biofilm effect of UV-C was evaluated on stainless steel discs contaminated with a 24 h-biofilm of each strain. Stainless steel discs measuring 6 mm in diameter and 3 mm in height were prepared by the ICAI School of Engineering, Pontificia Comillas University. The discs were sterilized using ethanol immersion followed by autoclaving (121°C, 15 min) before use. To perform the biofilm formation on the discs, they were placed into glass tubes containing 1 mL of bacteria or yeast suspension of each strain. The negative control was inoculated with only PBS. Tubes were incubated in an orbital shaker at 37°C for 24 h. After this period, discs were washed three times with PBS to remove nonadherent bacteria. Subsequently, the discs were placed on a stand and then the UV-C treatment was applied (UV-dose used: 228.6, 467.8, and 946.7 mJ/cm^2^) on each disc side. Once that UV-C treatment was completed, the discs were individually transferred to new glass tubes containing 1 mL PBS and sonicated for 10 min at 50–60 kHz to detach the biofilm. The sonicated suspension was then vortexed, one part was serially diluted, and 100 μL of dilution was cultured on a solid medium plate. All plates were incubated at 37°C for 24 h.

All data were expressed as log CFU/mL, and after the log_10_ reductions were calculated, considering as control the UV-untreated samples. All the experiments were replicated three independent times.

### 2.5 Log_10_ reduction calculation

To calculate Log_10_ reduction of microorganism growth determined by UV-C LEDs irradiation, the following Equation 1 was applied:


$$Log_{10}\;reduction=Log_{10}\left(A\right)-Log_{10}(B)$$ (1)

in which A was the Log_10_ microbial concentration in the contaminated not UV-C treated control, and B was the Log_10_ microbial concentration obtained after the UV-C disinfection at the different UV doses. The same calculation was used for both the *in vitro* setting and for the assay on stainless discs.

### 2.6 Determination of the D_90_ values and UV inactivation constant (*k*)

Based on the log_10_ reductions obtained, the UV doses causing a 90% reduction (i.e. 1 log_10_ reduction) of bacterial number (D_90_) were calculated with GraphPad Prism 8.0 (San Diego, CA, USA), by a non-linear regression curve model. From the D_90_ values, the UV inactivation constant (*k*) of each microorganism was calculated using Equation 2 (Mariita et al., 2022):


$$k\;(m^{2}/J)=\;\frac{-ln\;(1-0.9)}{D_{90}}$$ (2)

### 2.7 Statistical analysis

Statistical analyses were performed with GraphPad Prism 8.0 (San Diego, CA, USA), using a 2way ANOVA test, followed by Tukey's multiple comparison test. All the experiments and control conditions were performed in triplicates in three independent experiments. A difference was identified as significant at a *P* value of less than 0.05 (^*^), 0.01 (^**^), and 0.001 (^***^).

## 3 Results

In this study, the viability count was considered to measure the UV-C efficacy against both planktonic and sessile cells.

### 3.1 UV-C susceptibility of each tested strain in the planktonic stage

**Figure 1:**
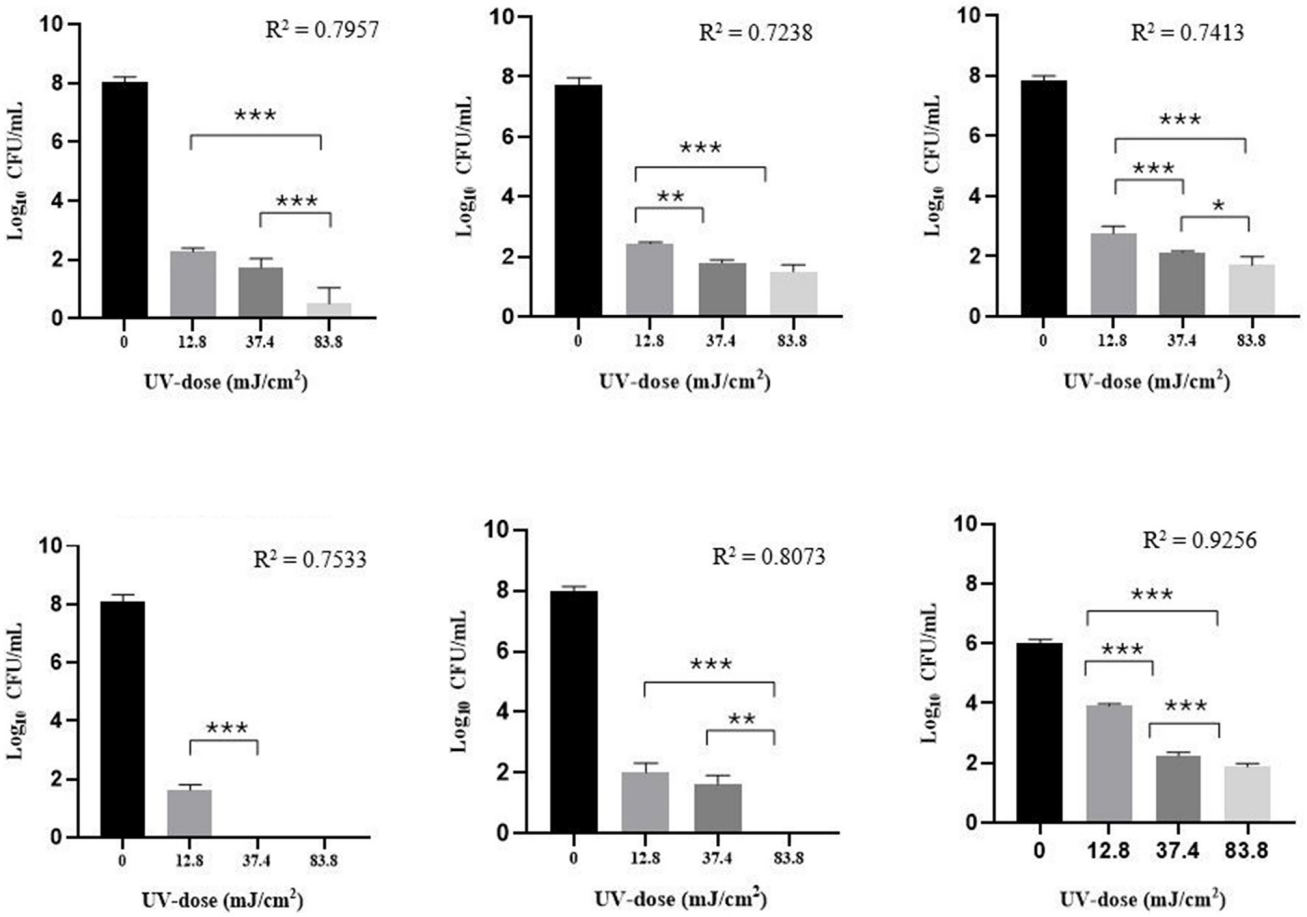
Log_10_ microbial load (CFU/mL) of MSSA, MRSA, MRSE, *E. coli, P. aeruginosa*, and *C. albicans* strains obtained onto solid medium applying a UV-dose of 0 (control), 12.8, 37.4, and 83.8 mJ/cm^2^. Bar charts are shown as mean ± standard deviation (SD). Mean values ± SD from three independent experiments (*n* = 3, for each experimental condition) (3 independent experiments). 2way ANOVA, *p < 0.05; **p < 0.01; ***p < 0.001.

To assess the intrinsic UV susceptibility of test microorganisms in the planktonic state, UV-C exposure tests were conducted by exposing microbial cells to different UV doses on solid agar medium. In this study phase, given the greater susceptibility of planktonic cells to UV than sessile cells, and because the objective was to evaluate the inactivation constant *k*, the following UV doses were used: 12.8, 37.4, and 83.8 mJ/cm^2^. The colony plate count confirmed the expected starting concentration of each microbial suspension and allowed us to calculate the microbial titers in the control plates. In the control plate without the UV-C treatment (0 mJ/cm^2^), the microbial titer of each tested strain was: 1.20 ± 0.45 x 10^8^ (MSSA), 5.9 ± 3.21 x 10^7^ (MRSA), 7.41 ± 2.71 x 10^7^ (MRSE), 1.43 ± 0.77 x 10^8^ (*E. coli*), 1.03 ± 0.43 x 10^8^ (*P. aeruginosa*), and 1.10 ± 0.34 x 10^6^ (*C. albicans*) CFU/mL. All preselected microbial strains demonstrated at least a 5 log_10_ decrease in viability after an irradiation of 12.8 mJ/cm^2^, except for *C. albicans*. Although the initial microbial titer of *C. albicans* was about 2 log lower than the other bacterial strains, a log_10_ reduction of 2.11 ± 0.09 was achieved by applying a UV dose of 12.8 mJ/cm^2^. A complete microbial inactivation (< 1 CFU/mL) was achieved only against *E. coli* and *P. aeruginosa* by applying a UV dose of 37.4 and 83.8 mJ/cm^2^, respectively (Figure 1). Comparing Gram positive and negative bacteria in the planktonic state, at the same UV dose, a significant difference (*p* < 0.01) in their susceptibility to UV-C was found. It was not possible to evaluate the constant *k* of *E. coli* through the non-linear regression curve since with two of the three UV doses used a titer < 1 CFU/mL was reached and the program could not extrapolate the D_90_ value (Table 2).

**Table 2:** Log_10_ reduction ± standard deviation (SD) (CFU/mL) and D_90_ ± SD of each tested strain, in planktonic culture, in relation to the UV dose used, and mean UV Inactivation Constant (*k*) obtained.

**Microbial strain**	**UV dose (mJ/cm^2^)**	**Log_10_ reduction ±SD (CFU/mL)**	**D_90_ ±SD (mJ/cm^2^)**	**Mean *k* (m^2^/J)**
MSSA ATCC 29213	12.8	5.70 ± 0.12	1.83 ± 1.57	0.126
	37.4	6.36 ± 0.39		
	83.8	7.58 ± 0.57		
MRSA ATCC 43300	12.8	5.31 ± 0.19	2.70 ± 1.86	0.085
	37.4	5.94 ± 0.24		
	83.8	6.25 ± 0.46		
MRSE^*^	12.8	5.06 ± 0.12	2.52 ± 1.43	0.091
	37.4	5.68 ± 0.13		
	83.8	6.11 ± 0.23		
*E. coli* ATCC 25922	12.8	6.48 ± 0.18	n.e	n.e
	37.4	8.15 ± 0.00		
	83.8	8.15 ± 0.00		
*P. aeruginosa* ATCC 15442	12.8	5.98 ± 0.40	1.89 ± 1.68	0.122
	37.4	6.35 ± 0.38		
	83.8	7.98 ± 0.09		
*C. albicans* ATCC 14053	12.8	2.11 ± 0.09	4.37 ± 1.09	0.053
	37.4	3.78 ± 0.12		
	83.8	4.14 ± 0.07		

Mean values ± SD from three independent experiments (*n* = 3, for each experimental condition) (3 independent experiments).

^*^clinical strain. n.e, not evaluable.

### 3.2 UV-C susceptibility of each tested strain in a 24 h-biofilm stage

**Figure 2:**
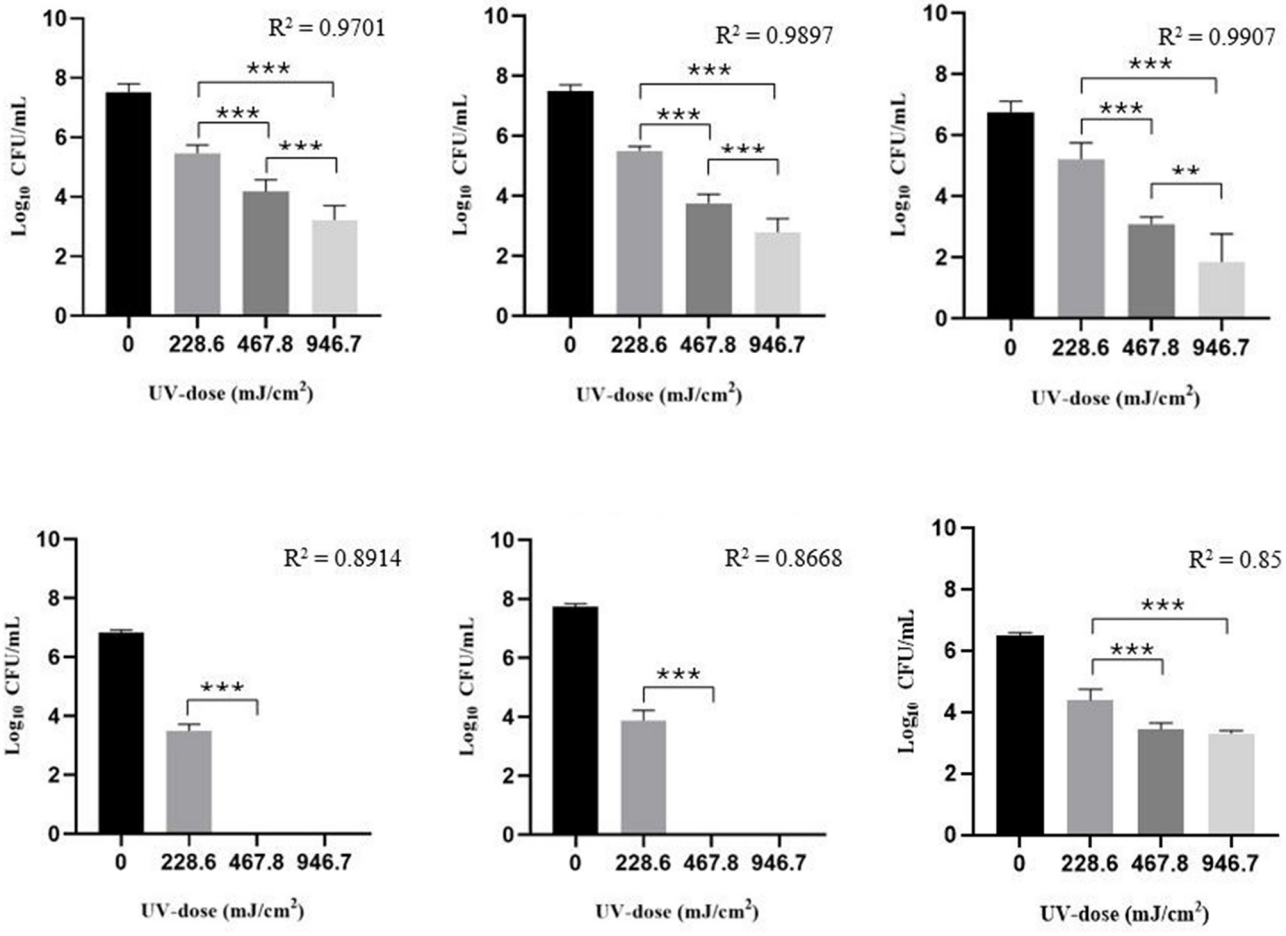
Survival of MSSA, MRSA, MRSE, *E. coli, P. aeruginosa*, and *C. albicans* cells grown in a 24 h-biofilm after exposure to UV-C irradiation applying a UV-dose of 0 (control), 228.6, 467.8, and 946.7 mJ/cm^2^. The tests were performed on dry biofilm in 96-well cell culture microtiter plates. Bar charts are shown as mean ± standard deviation (SD) from three independent experiments (*n* = 3, for each experimental condition) (3 independent experiments). 2way ANOVA, ***p < 0.001.

Following UV-C treatment a high reduction in viability was also found in the same strains grown in biofilms. Considering the initial cell suspension (0.5 and 0.35 McFarland), MSSA, MRSA, *P. aeruginosa* and *C. albicans* showed greater ability to produce biofilms. As reported in Figure 2, the microbial load decreased linearly as the UV dose increased (*R*^2^ shown in Figure 2). The log_10_ reduction values for each test microorganism are reported in Table 3.

**Table 3:** Log_10_ reduction ± standard deviation (SD) (CFU/mL) and D_90_ ± SD of each tested strain 24 h-biofilm in relation to the UV dose used, and mean UV Inactivation Constant (*k*) obtained.

**Test strain**	**UV dose (mJ/cm^2^)**	**Log_10_ reduction ±SD (CFU/mL)**	**D_90_ ±SD (mJ/cm^2^)**	**Mean *k* (m^2^/J)**
MSSA ATCC 29213	228.6	1.99 ± 0.33	67.12 ± 1.31	0.003
	467.8	3.27 ± 0.55		
	946.7	4.34 ± 0.70		
MRSA ATCC 43300	228.6	2.16 ± 0.41	87.20 ± 1.21	0.003
	467.8	3.77 ± 0.44		
	946.7	4.70 ± 0.60		
MRSE^*^	228.6	1.54 ± 0.32	129.1 ± 1.16	0.002
	467.8	3.58 ± 0.29		
	946.7	4.85 ± 0.98		
*E. coli* ATCC 25922	228.6	3.23 ± 0.07	n.e	n.e
	467.8	6.83 ± 0.07		
	946.7	6.83 ± 0.07		
*P. aeruginosa* ATCC 15442	228.6	3.84 ± 0.42	n.e	n.e
	467.8	7.72 ± 0.10		
	946.7	7.72 ± 0.10		
*C. albicans* ATCC 14053	228.6	2.10 ± 0.41	85.11 ± 3.06	0.003
	467.8	2.73 ± 0.60		
	946.7	3.17 ± 0.08		

Mean values ± SD from three independent experiments (*n* = 3, for each experimental condition) (3 independent experiments).

^*^clinical strain. n.e, not evaluable.

Compared with the planktonic form, microbial cells grown in 24 h-mature biofilm showed greater UV-C resistance. Despite this, a reduction of at least 2 logs was achieved by applying a UV dose of 228.6 mJ/cm^2^, except for MSSE, which in a 24 h-biofilm showed a higher UV-C resistance. The different UV susceptibility between Gram positive and Gram negative emerged more in this phase of the study, as with both *E. coli* and *P. aeruginosa* biofilms a < 1 CFU/mL was achieved by applying a UV dose of 467.8 mJ/cm^2^. Considering the two Gram-negative strains, in a biofilm state *P. aeruginosa* was slightly more susceptible than *E. coli*. Similarly, there was a progressive reduction in the viability of MRSA and MSSA biofilms as the UV dose increased. Even in this case, it was not possible to evaluate the *k* constant of all tested microorganisms.

### 3.3 Efficacy of UV-C against biofilm on stainless steel discs

**Figure 3:**
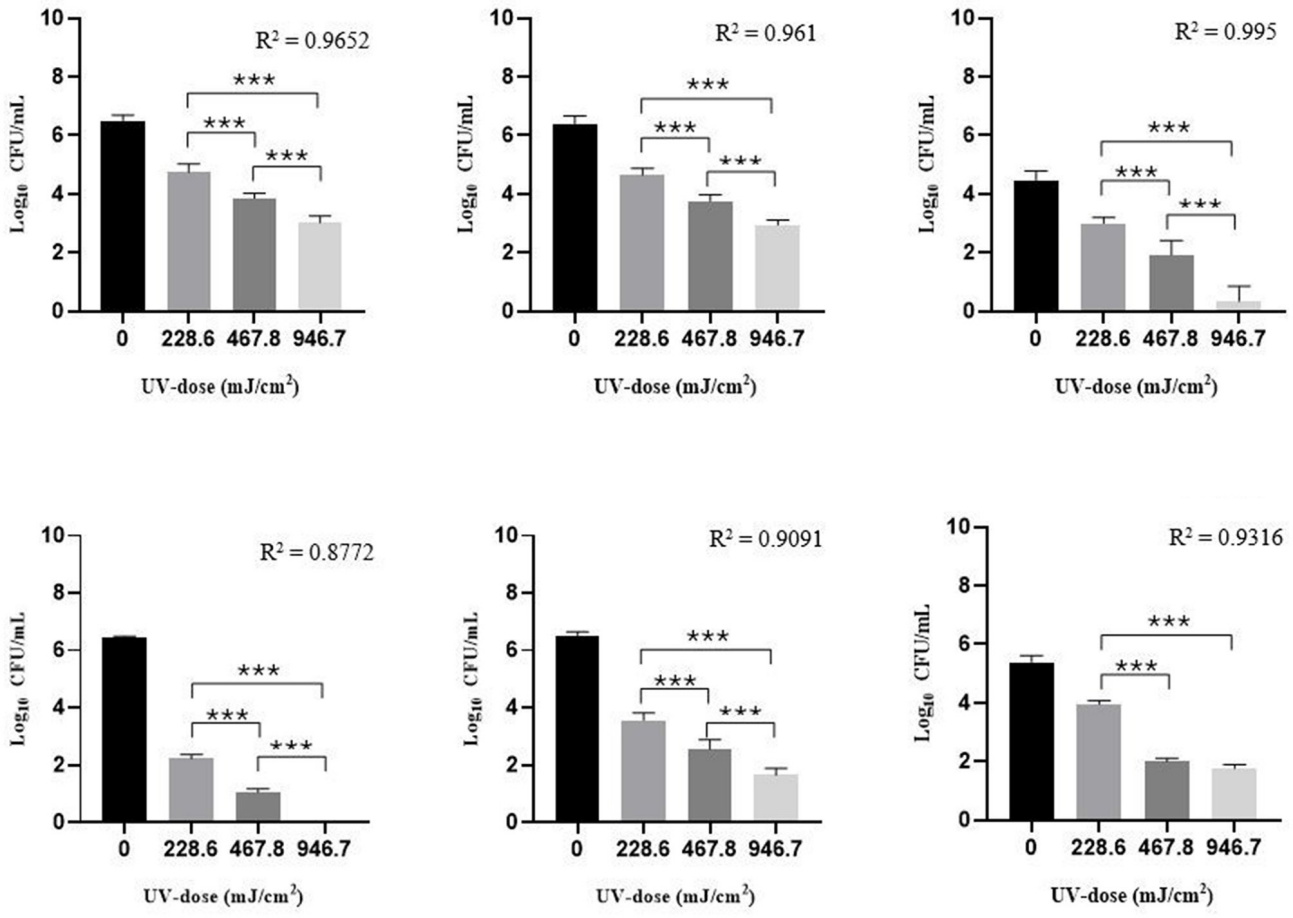
Survival of MSSA, MRSA, MRSE, *E. coli, P. aeruginosa*, and *C. albicans* cells grown in a 24h-biofilm on stainless steel discs after exposure to UV-C irradiation applying a UV-dose of 0 (control), 228.6, 467.8, and 946.7 mJ/cm^2^ on each disc side. Bar charts are shown as mean ± standard deviation (SD) from three independent experiments (*n* = 3, for each experimental condition) (3 independent experiments). 2way ANOVA, **p < 0.01; ***p < 0.001.

In addition to performing log_10_ reduction evaluations on plate biofilms, tests were also performed on stainless steel discs. The objective was to evaluate the anti-biofilm efficacy of UV-C irradiation even on a surface particularly common in the hospital setting, as stainless steel, contaminated with a 24 h-biofilm. In this second phase of the study, it was found that a complete microbial inactivation (< 1 CFU/mL) was achieved only by exposing discs contaminated with 24 h-biofilms of *E. coli* to a UV dose of 946.7 mJ/cm^2^ (Figure 3).

In general, all microorganisms grown in biofilms showed greater resistance to UV disinfection when located on stainless steel surfaces (Table 4). Compared to other tested microorganisms, the clinical MRSE strain showed a lower capacity to produce biofilm on stainless steel discs. Considering the lowest UV dose (228.6 mJ/cm^2^), a 2 log_10_ reduction was achieved only with the two Gram-negative bacteria considered in the study. In general, as the UV dose applied increased, an increase in log_10_ reduction was observed in a linear manner in all biofilms of the tested microorganisms.

**Table 4:** Log_10_ reduction ± standard deviation (SD) (CFU/mL) and D_90_ ± SD of each tested strain 24 h-biofilm on stainless steel discs in relation to the UV dose used, and mean UV Inactivation Constant (*k*) obtained.

**Test strain**	**UV dose (mJ/cm^2^)**	**Log_10_ reduction ±SD (CFU/mL)**	**D_90_ ±SD (mJ/cm^2^)**	**Mean *k* (m^2^/J)**
MSSA ATCC 29213	228.6	1.88 ± 0.28	54.97 ± 1.40	0.004
	467.8	2.78 ± 0.16		
	946.7	3.34 ± 0.35		
MRSA ATCC 43300	228.6	1.92 ± 0.11	53.11 ± 1.33	0.004
	467.8	2.80 ± 0.30		
	946.7	3.27 ± 0.14		
MRSE^*^	228.6	1.21 ± 0.20	195.3 ± 3.37	0.001
	467.8	2.29 ± 0.49		
	946.7	3.88 ± 0.52		
*E. coli* ATCC 25922	228.6	4.22 ± 0.13	19.66 ± 1.17	0.012
	467.8	5.39 ± 0.14		
	946.7	6.44 ± 0.03		
*P. aeruginosa* ATCC 15442	228.6	2.96 ± 0.22	25.59 ± 1.49	0.009
	467.8	3.96 ± 0.42		
	946.7	4.87 ± 0.26		
*C. albicans* ATCC 14053	228.6	1.43 ± 0.16	150.4 ± 1.05	0.002
	467.8	3.38 ± 0.19		
	946.7	3.62 ± 0.18		

Mean values ± SD from three independent experiments (*n* = 3, for each experimental condition) (3 independent experiments).

^*^clinical strain.

## 4 Discussion

Nowadays, HAIs represent a challenge in the management of hospital environments, within which there are frail people who are more susceptible to contracting an infection. These infections, which are often due to the presence of antibiotic-resistant bacteria, can spread rapidly in these indoor environments through surfaces, medical devices, and contact between healthcare personnel and patients. The presence of bacterial biofilms significantly increases the risk of HAI, given their increased resistance to the most common environmental disinfection techniques. The need to find effective solutions to reduce the spread of pathogens in hospital environments is further accentuated by the need to adopt more sustainable and eco-friendly disinfection practices. The use and development of new UV-C-based technologies could represent an effective solution to guarantee the healthiness of hospital environments.

There are only a few reports in the literature that study the inactivation of sessile bacterial cells using UV-C devices, and those few that do exist often do not deal with pre-formed biofilms. Moreover, its use against the biofilm of common microorganisms causing HAI has not been properly assessed. Considering that, we performed this 2-phase study where we evaluated the anti-biofilm effect of UV-C (254 nm) irradiation against preformed biofilms of some of the microorganisms most involved in HAIs.

For this purpose, biofilm-producing microorganisms of hospital interest were considered in this study. Infections caused by *S. aureus* and *S. epidermidis* are among the most frequent causes of healthcare-associated infections, and also *S. aureus* is often related to antibiotic resistance in hospital settings (CDC and ICAN, 2019; Guo et al., 2020). Therefore, methicillin-susceptible and methicillin-resistant strains of *S. aureus* were considered in this study. *P. aeruginosa* is an opportunistic human pathogen that causes high morbidity and mortality in immunocompromised and hospitalized individuals (Antonelli et al., 2021). Lastly, *E.coli* and *Candida* spp. are often involved in catheter-related infection (Díaz-Navarro et al., 2023; Zou et al., 2023).

In the first phase of the study exposure tests to different UV doses of the tested microorganisms in a planktonic phase were performed on a solid culture medium. The objective of this phase was to evaluate the UV-C susceptibility of the test microorganisms. In this phase of the study, the UV doses used were 12.8, 37.4, and 83.8 mJ/cm^2^. Results obtained from irradiation tests on planktonic bacterial cells showed that MSSA, MRSA, and MRSE strains had a similar UV-C susceptibility, in which a log_10_ reduction >5 logs was already achieved at the lowest UV dose (12.8 mJ/cm^2^). *E. coli* proved to be the most susceptible to UV-C irradiation; in fact, by applying a UV dose of 12.8 mJ/cm^2^, the initial titer was reduced by 6.48 ± 0.18 logs, while at the same UV dose, a log_10_ reduction of 5.98 ± 0.40 was achieved with *P. aeruginosa*. In this case, there was no significant difference (*p* > 0.05) in UV-C susceptibility between Gram positive (MSSA, MRSA, and MRSE) and Gram negative (*E.coli* and *P. aeruginosa*) bacteria. However, the UV dose of 12.8 mJ/cm^2^ used was not sufficient to achieve a similar log_10_ reduction against *C. albicans*, in fact, a log_10_ reduction of 4.14 ± 0.07 was achieved by applying a UV dose of 83.8 mJ/cm^2^, thus proving to be the most resistant of the microorganisms tested in this work in the planktonic state. Similar log_10_ reduction values were obtained in the study by Duering et al. (2023), in which the researchers obtained a log_10_ reduction >6 log of planktonic *E. coli* and exposed to 20 mJ/cm^2^. In contrast, different results were obtained with *S. aureus* and *C. albicans*, with which the researchers obtained a greater reduction of >6 and >4 logs, respectively. It must be considered that these two microorganisms, differently from *E.coli*, were exposed to higher UV-C doses (1,500 mJ/cm^2^) than those used in this study, and the fact that the tests with these two microorganisms were performed only in PBS. As stated by the authors themselves, after air-drying, the bacteria settle and form a multilayer on the surface, which is difficult to penetrate with UV-C rays. Considering *E. coli* and *P. aeruginosa*, similar results were obtained by dos Santos and de Castro (2021) on planktonic cells spread on solid culture medium, but applying a UV dose of 912 mJ/cm^2^. Differently, it was with *S. epidermidis* and *S. aureus* strains with which researchers achieved a title < 1 Log CFU/mL. However, it should be considered that the single UV dose applied by dos Santos and de Castro (2021) in the planktonic phase was much higher than that used in our study against planktonic cells.

Subsequently, the UV-C susceptibility of the same microorganisms tested was evaluated in a biofilm state matured 24 h to assess how much higher their resistance to the disinfection process was. To our knowledge, there are only a few studies in which the UV-C susceptibility of preformed biofilms has been assessed. As is known from other disinfection methods, even when considering UV-C irradiation, microorganisms in a biofilm state were found to be more resistant to UV-C (Corrêa et al., 2017; de Carvalho, 2017). This greater resistance of sessile cells compared to planktonic cells was confirmed in the present work, in which all bacteria showed a higher *k* constant when enclosed in a biofilm. In this study, we observed that, in general, UV resistance increased about 30 times. Despite this higher UV resistance, the inactivation efficacy against all microbial-tested strains remains relatively stable at higher UV doses. Against biofilm, the UV doses used were 228.6, 467.8, and 946.7 mJ/cm^2^. Against Gram negative bacteria (*E. coli* and *P. aeruginosa*), a UV dose of 467.8 mJ/cm^2^ was enough to achieve a bacterial load < 1 CFU/mL, while for Gram positive bacteria (MSSA, MRSA, and MRSE) even the higher UV dose used (946.7 mJ/cm^2^) was not enough to achieve the same result (< 1 CFU/mL). In fact, in contrast to the results obtained from planktonic cells, Gram negative bacteria were found to be significantly (*p* < 0.05) more susceptible than Gram positive bacteria when in a biofilm state. Probably this difference in susceptibility did not emerge even in the planktonic phase since all microorganisms are much more sensitive to UV. As reported by Mutschlechner et al. (2024), the different susceptibility to UV irradiation among bacteria is mostly attributed to structural differences, particularly the thicker peptidoglycan layer in Gram positive bacteria, which reduces the penetration of UV into the cell and its genetic damage. The same authors also reported that the greater resistance of gram-positive bacteria may also be due to their ability to produce pigments capable of absorbing and dissipating UV radiation, as well as the presence of more efficient DNA repair mechanisms than gram-negative bacteria. Again, *C. albicans*, even applying a maximum UV-C dose (946.7 mJ/cm^2^), a log_10_ reduction of 3.17 ± 0.08 was achieved, confirming its high UV resistance. Considering *P. aeruginosa*, Argyraki et al. (2017) achieved log_10_ reduction values different from ours. The researchers applying a UV dose of 1,000 mJ/cm^2^ achieved a log_10_ reduction of 1 log only. This could be because, as also stated by Labadie et al. (2024), irradiation efficiency depends on initial cell density. Argyraki et al. (2017) before UV treatment had an initial bacterial titer > 9 log, while in our study we started from a *P. aeruginosa* initial bacterial titer of 7.72 ± 0.10 log_10_ CFU/mL.

In the second phase of the study, the anti-biofilm effectiveness of UV-C was evaluated on stainless steel discs contaminated with 24 h-biofilm of each strain. The choice of this test material was made since it represents one of the main materials in the hospital setting (e.g., surgical instruments, hospital beds, surgical room surface). Although the disinfection efficacy of UV irradiation was also high in this phase of the study, differences in microbial inactivation emerged compared with previous *in vitro* biofilm tests. It must be considered that the edges of the discs were not well exposed to UV-C rays, so UV efficacy may be underestimated. In addition, as reported in the literature, it should also be considered that the type of surface treated can influence the disinfection effectiveness of UV-C (Kim and Kang, 2020; von Hertwig et al., 2023). Despite this, a log_10_ reduction >3.27 ± 0.14 was achieved on the stainless steel discs with all Gram positive bacteria with a UV dose of 946.7 mJ/cm^2^. Against Gram negative bacteria a log_10_ reduction up to 6.44 ± 0.03 was achieved, reconfirming the increased susceptibility of Gram negatives to UV-C irradiation. Considering *C. albicans*, similar results were obtained in the study of Binns et al. (2020) in which they irradiated a *C. albicans* biofilm on a Poly(methylmethacrylate) Resin (PMMA) surface applying a UV dose of 210 mJ/cm^2^. Comparing it with our results, their data were similar to those obtained with a UV dose of 228.6 mJ/cm^2^ on plate-irradiated biofilms (log_10_ reduction 2.10 ± 0.41 CFU/mL) but different from those obtained on stainless steel discs (log_10_ reduction 1.43 ± 0.16 CFU/mL). These differences could be due to the surface material being able to influence the effectiveness of UV-C irradiation.

It must be emphasised that when applying a UV disinfection treatment on a biofilm, several factors (e.g., UV-dose, type of microorganism, type of surface) may influence its effectiveness, determining whether the effect will be bactericidal or bacteriostatic. As reported by Marasini et al. (2025), the thickness of the biofilm may impact the effectiveness of UV-C irradiation, which may have a bactericidal effect on bacteria in the superficial layers of the biofilm, but a bacteriostatic effect on bacteria in the deeper layers. This is due to reduced UV penetration through the extracellular matrix of the biofilm, which can absorb and disperse UV radiation, reducing its effectiveness in the lower layers. A valid solution could be to use multiple wavelengths or pulsed UV devices, which can have a greater penetration efficacy, use longer and repeated UV exposure times, and pre-cleaning of surfaces to remove organic material and reduce the thickness of biofilm.

It should be noted that the initial microbial concentration used in the work did not reflect that usually found in a hospital setting because it is much higher than those generally found in the nosocomial environment (Messina et al., 2013; Evangelista et al., 2015). In this study, the concentration of the starting microbial inoculum was selected exclusively to reproduce the method described in previous studies for the formation of a biofilm *in vitro*, and not to simulate a real-world contamination level (Díaz-Navarro et al., 2023; Márquez-Gómez et al., 2023). As reported in a previous study, the initial microbial concentration can influence the UV-C efficacy in microbial inactivation, reducing the bactericidal efficacy of UV as the microbial titre increases (Palma et al., 2025). However, despite the high initial microbial titer concentrations, equally optimal microbial inactivation was achieved with all tested microorganisms. This suggests that in a real-life context, the effectiveness of the lamp might be higher.

As reported in other studies (Knobling et al., 2023b; Siwe et al., 2024), to date, there are still no European or international standards for UV-based device efficacy evaluation regarding the various pathogens' inactivation on surfaces in hospital environments, with the exception of the standard BS 8628:2022 (British Standard Institution, 2022). Comparing our results with this standard, the Log_10_ reductions obtained on planktonic cells were higher than the threshold value reported in BS 8628:2022. It should be pointed out that biofilms are not mentioned in this standard, and to our knowledge, there are no UV inactivation threshold values available for them. This underlines the need for standards that consider the efficacy of UV inactivation on sessile cells, as they are a major issue in hospital environments.

It must be considered that five of the six microbial strains tested were standard ATCC strains. As reported by Knobling et al. (2023a), clinical isolated strains may show increased resistance against the bactericidal effect of UV irradiation. Moreover, the biofilm maturity state and different microbial species within it can further influence the disinfection effectiveness of UV (von Hertwig et al., 2023; Jahid et al., 2014). Therefore, further studies considering older biofilms, mixed-species biofilms, and clinical isolated microorganisms will be carried out.

Another limitation of this study is that the tests were performed in the absence of organic soil load. Zwicker et al. (2022) demonstrated how the germicidal efficacy of UV can be affected by the presence of organic soil load on surfaces, testing the radiation depth penetration and the microbial inactivation of different wavelengths using organic matter such as albumin, artificial sweat, and artificial wound exudate. The authors reported that a wavelength of 222 nm showed a reduced depth penetration ability and consequently a reduced microbial inactivation compared to higher wavelengths (from 254 nm), where the depth penetration becomes comparable to that observed with NaCl solution. In light of this, to optimize disinfection processes, a disinfection treatment should be carried out on surfaces that have been previously deterged. Lastly, it must be considered that the physiological and genetic consequences of UV-surviving cells were not evaluated in this study. In particular, we did not assess whether the surviving microbial cells showed greater tolerance to subsequent UV exposure or a greater ability to rebuild biofilms. Given that UV treatment can induce DNA damage and potentially increase genetic diversity through mutagenesis, future studies should evaluate the long-term consequences of UV exposure on microbial adaptation and resistance development (Krishna et al., 2007).

To our knowledge, this was the first time that the *k* inactivation constants were reported within a study on biofilms, comparing them with the same microorganisms in the planktonic phase as well as on surfaces. Furthermore, there are no ISO standards in which surface *k* constants are reported. Knowledge of the surface *k* of a microorganism of hospital interest, such as those considered in this study, is critical to create disinfection protocols that include UV-C irradiation in hospital environments. According to our results, the application of UV-C irradiation could be a valid additional method in the management of biofilm-related HAI in hospital environments.

In addition to its use for environmental sanitation purposes, the application of UV-C technology in a clinical setting could also be a promising solution regarding the disinfection of biomaterials and prostheses colonized by infections that cannot be immediately removed (e.g., periprosthetic infections when a Debridement, Antibiotics and Implant Retention, or DAIR, is being approached) or as a prophylactic approach before implant insertion. Only 3 studies have reported preliminary *in vitro* findings. Janson et al. (2017) tested a combination of TiO_2_ and H_2_O_2_ under UV light as an alternative method for disinfection of dentures and dental implants. Promising results regarding the application of UV-C in preserving and maintaining the healthiness of silicone-facial prosthetic material were reported by Malateaux et al. (2021, 2024). Considering this, future studies evaluating the application of UV irradiation in a clinical setting are needed, to reduce the risk of HAI.

## 5 Conclusion

The application of UV-C irradiation could be a valid additional approach in the management of biofilm HAI. The UV-C efficacy was very high in all three principal experimental conditions of this study, i.e., both in the planktonic phase, in the biofilm phase, and on a stainless steel surface contaminated with a biofilm. The results of this study indicate that UV-C irradiation has a high potential in the microbial biofilm inactivation of some of the most prevalent microorganisms in hospital environments. In the future, other microorganisms of hospital interest will be tested, and other surfaces will need to be considered.

## Data Availability

The original contributions presented in the study are included in the article/supplementary material, further inquiries can be directed to the corresponding author.
