# Correction: Assessment of the anti-biofilm effect of UV-C irradiation (254 nm) against healthcare associated infections related microorganisms

**DOI:** 10.3389/fmicb.2025.1741013

**Published:** 2025-12-09

**Authors:** Francesco Palma, Marta Díaz-Navarro, Andrés Visedo, Pablo Sanz-Ruíz, Giorgio Brandi, Giuditta Fiorella Schiavano, María Guembe

**Affiliations:** 1 Department of Biomolecular Sciences, University of Urbino Carlo Bo, Urbino, Italy; 2 Instituto de Investigación Sanitaria Gregorio Marañón, Madrid, Spain; 3 Department of Clinical Microbiology and Infectious Diseases, Hospital General Universitario Gregorio Marañón, Madrid, Spain; 4 Medicine Department, School of Medicine, Universidad Complutense de Madrid, Madrid, Spain; 5 Department of Orthopaedic Surgery and Traumatology, Hospital General Universitario Gregorio Marañón, Madrid, Spain; 6 Department of Humanities, University of Urbino Carlo Bo, Urbino, Italy

**Keywords:** UV-C, biofilm, hospital surface, disinfection, healthcare-associated infection

**Figures 2 and 3** were in the wrong order in the PDF and HTML version of this paper.

The images, not the captions, of Figures 2, 3 were incorrect.

An incorrect **Funding** statement was provided. The correct funding statement reads:

“The author(s) declare that financial support was received for the research and/or publication of this article. The open access publication of this paper was funded by research grants from the Department of Humanities, DISTUM of the University of Urbino Carlo Bo.”

An **Acknowledgement** section was not included. The Acknowledgement statement is below:

“The authors disclosed receipt of the following financial support for publication of this article from the Department of Humanities – DISTUM of the University of Urbino Carlo Bo.”

The original version of this article has been updated.

